# Bidirectional Association Between Knee Osteoarthritis and Depressive Symptoms: A Nationwide Cohort Study

**DOI:** 10.1093/geroni/igaa057.710

**Published:** 2020-12-16

**Authors:** Han Lu, Shaomei Shang, Limin Wang, Hongbo Chen

**Affiliations:** Peking University, Beijing, China

## Abstract

Both knee osteoarthritis (KOA) and depressive symptoms are common health issues affecting the quality of life of old adults. Although it is presumed that KOA has a bidirectional relationship with the depressive symptoms, no cohort study has proven it. This is the first study to determine the strength of association for the bidirectional relationship between KOA and depressive symptoms. Data were gathered from the nationally survey of China Health and Retirement Longitudinal Study in 2011-2015. The presence of depressive symptoms was defined by the 10-item Center for Epidemiologic Studies Depression Scale score of 10 or higher. The adjusted Cox proportional hazards regression model was conducted to estimate hazards ratios (HRs). Controlled covariates include gender, age, education, marital status, residence, number of chronic diseases, and disability. The analysis of KOA predicting the depressive symptoms onset consisted of 4,377 participants free from depressive symptoms at baseline. During 4 years follow-up, diagnosed KOA participants were more likely to have depressive symptoms than their peers without KOA (HR = 1.50, 95% CI: 1.23-1.83). The parallel analysis of depressive symptoms predicting KOA onset included 6,848 participants without KOA at baseline, those with depressive symptoms had a higher relative risk of developing KOA (HR = 1.64, 95% CI: 1.41-1.92). Our results provide compelling evidence that the KOA-depressive symptoms association is bidirectional, highlighting the importance of evaluating the relationship between physical and mental health among older people. Particularly, taking this association into consideration in the risk assessment and primary prevention of KOA and depression symptoms.

